# Epidural myelolipoma in a Husky-cross: a case report

**DOI:** 10.1186/1751-0147-55-28

**Published:** 2013-04-04

**Authors:** Marina Verena Hoffmann, Davina Claudia Ludwig, Charlotte Lempp, Verena Haist, Veronika Maria Stein

**Affiliations:** 1 Department of Small Animal Medicine and Surgery, University of Veterinary Medicine Hannover, Buenteweg 9, D-30559, Hannover, Germany; 2 Department of Pathology, University of Veterinary Medicine Hannover, Buenteweg 17, Hannover, D-30559, Germany

**Keywords:** Haematopoietic proliferation, Sled dog, SPAIR

## Abstract

Epidural spinal myelolipoma was diagnosed in an 11.5-year-old castrated male Husky-cross that was evaluated at the veterinary teaching hospital due to progressive thoracolumbar spinal hyperaesthesia and mild proprioceptive pelvic limb ataxia. A focal, ill-defined mildly inhomogenous extradural mass lesion was detected by MRI. The dog was euthanized. At necropsy an extradurally located reddish mass of about 2.5 cm in diameter was present in the vertebral canal. The mass was identified histopathologically as an epidural myelolipoma.

## Background

Myelolipomas are benign tumors consisting of mature fat interspersed with haematopoietic elements resembling bone marrow. Extraadrenal myelolipomas are suspected to arise from uncommitted mesenchymal cells. Their occurrence is thought to be influenced by haematopoietic growths factors, which mediate the recruitment of pluripotent haematopoietic stem cells [1]. They are rare findings in almost all domestic animals and are detected occasionally in the spleen and liver of aged dogs and cats [2]. In cattle and dogs, they occur infrequently as incidental findings in the adrenal gland [3,4].

In most cases there is no clinical relevance of this haematopoietic proliferation, but large tumors may cause clinical signs as a result of a mass effect [5]. Albeit listed as a tumor in the current WHO-classification, the classification of myelolipomas is under intense debate as it is not clear whether they represent real neoplasias, or rather ectopic proliferations, hamartomas or choristomas [2].

Epidural myelolipomas have previously been described in a Siberian husky [6] and in an Alaskan Malamute [7]. The present report describes the third case of epidural spinal myelolipoma in a male sled dog. The diagnosis was supported by MRI including a fat suppression sequence and confirmed by histopathology.

## Case presentation

An 11.5-year-old castrated male Husky-cross was evaluated because of a 3 week history of thoracolumbar spinal hyperaesthesia. Treatment with phenylbutazone and prednisolone for 2 weeks by the referring veterinarian did not improve clinical signs.

At the time of initial admission to the veterinary teaching hospital, general physical examination only confirmed the thoracolumbar spinal hyperaesthesia. Haematological parameters and radiographs of the vertebral column were unremarkable. Treatment with gabapentin and metamizol (Pyrazolone derivative, Non-Steroidal Anti-inflammatory Drug) was initiated.

**Figure 1:**
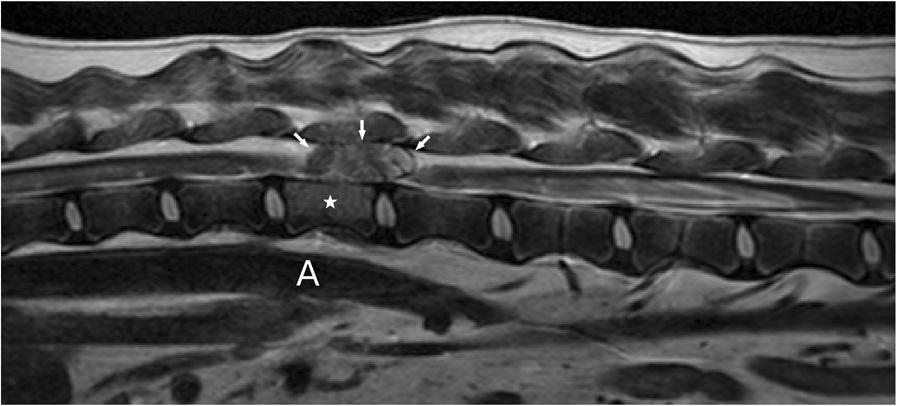
T2-weighted sagittal MR image (cranial is to the left) showing an iso- to hyperintense epidural mass (arrows) compressing the spinal cord at the level of L1 (star), for orientation aorta (A).

**Figure 2:**
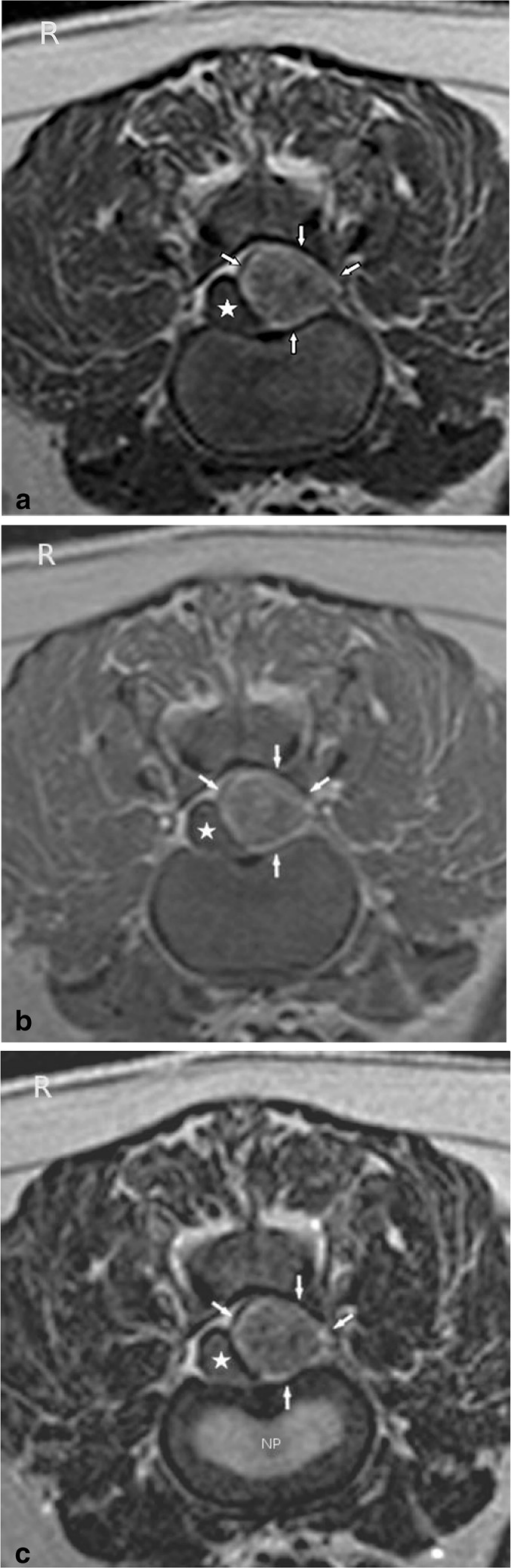
**a: T1-weighted transverse MR image (without contrast) at the level of the L1/L2 intervertebral disc space. **Notice the predominantely hyperintense signal of the mass (arrows) with areas of mixed intensity. The mass is displacing the spinal cord (star) to the right. **b: **T1-weighted transverse MR image (post contrast) at the level of the L1/L2 intervertebral disc space. Notice the predominantely hyperintense signal of the mass (arrows) with areas of mixed intensity. The mass is displacing the spinal cord (star) to the right and does not contrast enhance. **c: **T2-weighted transverse MR image at the level of the L1/L2 intervertebral disc space. Notice the predominantely hyperintense signal of the mass (arrows) compared to spinal cord parenchyma and the hypointense signal compared to the nucleus pulposus (NP). The mass has areas of mixed intensity and is displacing the spinal cord (star) to the right.

**Figure 3:**
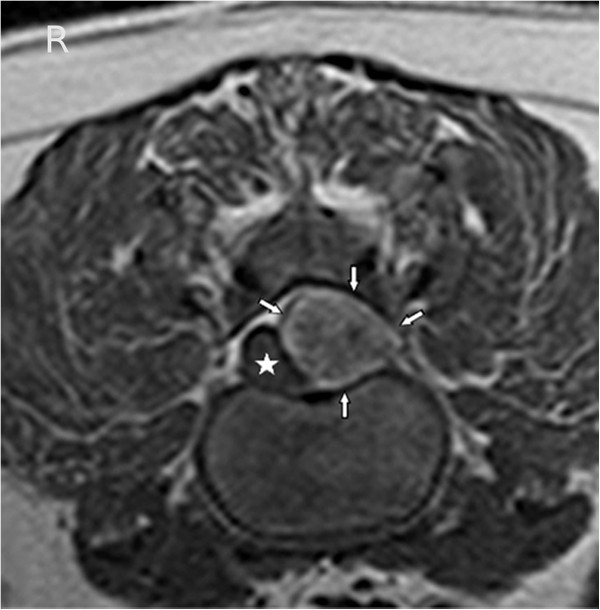
**SPAIR sequence transverse MR image at the level of the L1/L2 intervertebral disc space.** Notice the hypointense lesion (arrows) compared to the spinal cord (star) and nucleus pulposus (NP).

Three days after initial presentation, the hyperaesthesia progressively worsened and the dog was presented again. As compared to initial presentation, the neurological examination revealed that the thoracolumbar spinal hyperaesthesia had progressed and was severe. Additional mild proprioceptive ataxia in the pelvic limbs was consistent with a T3-L3 spinal cord segments localisation. MRI was obtained under general anesthesia using a 3.0 tesla scanner (Achieva 3.0T, Philips Medizin Systeme GmbH, D-22335 Hamburg, Germany) (Figures 1, 2, 3). Anesthesia was induced with levomethadone, diazepam and propofol and maintained by administration of isoflurane in oxygen-nitrous oxide. Results of CSF-analysis obtained via lumbar puncture were unremarkable.

T2-weighted sagittal MR images showed a heterogenous epidural mass, hyperintense compared to spinal cord parenchyma, but hypointense compared to CSF/epidural fat signal. The mass was extending from the cranial aspect of the first lumbar vertebral body to the cranial aspect of the second lumbar vertebral body and compressing the spinal cord (Figure 1). The T1-weighted transverse MR images (without contrast) revealed an extradural left sided lesion compressing the spinal cord to the right side (Figure 2a) There was no enhancement after administration of Gadolinium based paramagnetic contrast medium (Figure 2b).This mass was hyperintense to the spinal cord on T1- and T2- weighted images, hyperintense compared to the nucleus pulposus in transverse T1-weighted images (Figure 2a) and hypointense compared to the nucleus pulposus in transverse T2-weighted images (Figure 2c), both with moderate degree of heterogeneity. The mass was hypointense compared to spinal cord parenchyma on a fat suppression sequence (Spectral Adiabatic Inversion Recovery sequence) (Figure 3). The vertebral body of L1 seemed mildly hyperintense on T2-weighted images (Figure 1) and was hypointense in SPAIR. Because of the hyperintensity in T1- and T2- weighted images compared to spinal cord parenchyma and the hypointensity in SPAIR, the lesion was suspected to be primarily composed of fat tissue.

Differential diagnoses for the MRI findings of this dog were idiopathic epidural fat inflammation, epidural lipoma and epidural myelolipoma.

**Figure 4:**
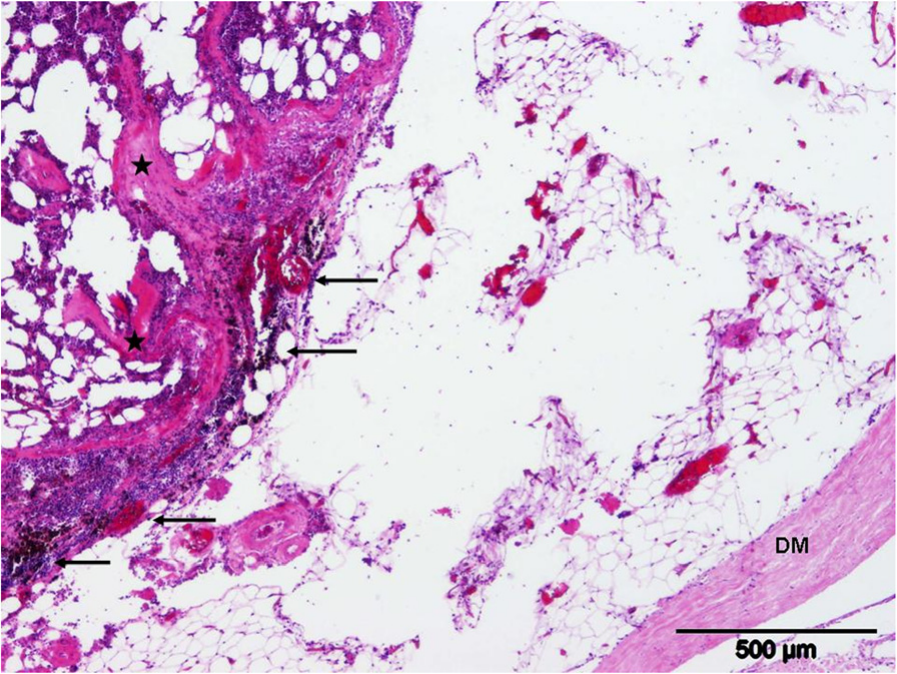
**Dura mater (DM) of the lumbar spinal cord with the adjacent mass consisting of adipocytes and haematopoietic cells with fibrous connective tissue (stars). **Perifocally, acute haemorrhage and haemosiderophagia are evident (arrows). Hematoxylin and Eosin staining.

**Figure 5:**
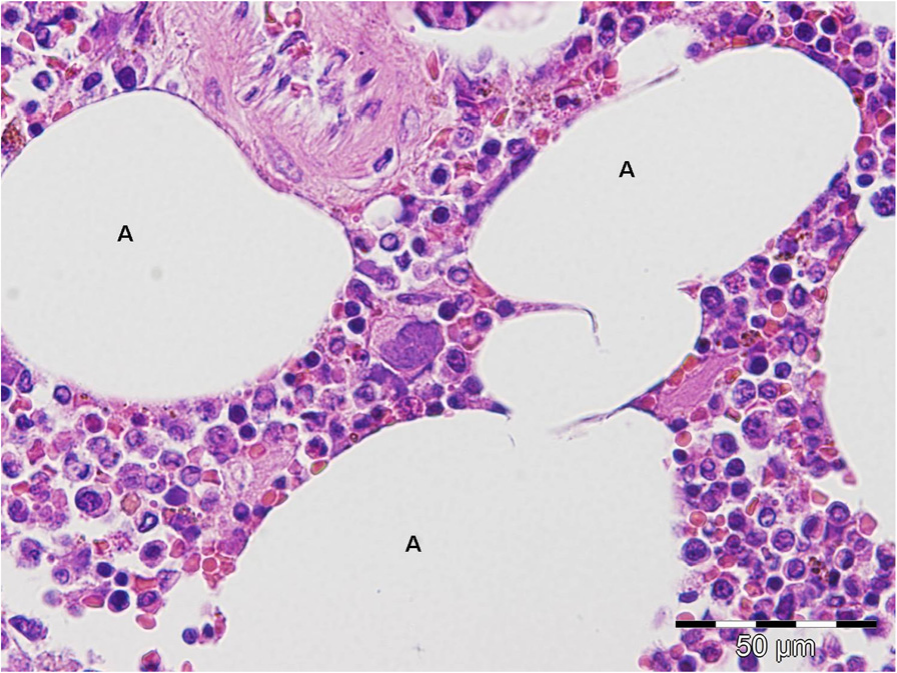
**Higher magnification of the mass consisting of mature adipocytes (A) and haematopoietic cells of the myeloid, erythroid and megakaryocytic lineage. **Hematoxylin and Eosin staining.

Based on the MRI findings, the progression of clinical signs and the unknown prognosis the owners elected euthanasia. At necropsy, an extradurally located reddish mass of about 2.5 cm in diameter was present in the vertebral canal at the level of the vertebrae L1 to L2. There were no morphological changes of the vertebral body. Histologically, the mass in the vertebral canal was poorly demarcated by a discontinuous fibrous capsule and consisted predominantly of mature adipocytes and cell rich areas. The latter were composed of haematopoietic cells of the myeloid, erythroid and megakaryocytic lineage in proportions equivalent to normal mature bone marrow (Figures 4 &5). In addition, the mass contained multiple small bony spicules as well as fibrous septae. Acute haemorrhage and haemosiderophagia were evident, predominantly in the periphery of the mass. The corresponding spinal cord segments as well as adjacent parts caudally showed moderate degenerative lesions consisting of dilated myelin sheaths, spheroid formations, and myelinophagia. These findings led to the diagnosis of an epidural myelolipoma.

MRI was especially useful in determining the underlying cause of the clinical manifestations and source of the dog´s thoracolumbar spinal hyperaesthesia and proprioceptive ataxia. Particularly the fat suppression sequence, because the hypointensity seen on this sequence is most compatible with the lesion being predominantly composed of adipose tissue, lead to the suspicion, that the diagnosis could be lipoma or myelolipoma. SPAIR is chemically selective and in comparison to a chemically non-selective fat suppression technique such as STIR (short T1 inversion recovery) only the fatty tissue is inverted, which is an advantage of this technique. It is useful in cases where water-based tissue lesions may have a T1 similar to that of lipids, because the non-selective scheme of STIR, in which the inversion pulse affects all tissues, can provide misleading results. Myelolipoma (*myelo-*, for marrow, *lipo* for fat and *oma* meaning tumor or mass) is a benign tumor-like lesion composed of mature adipose (fat) tissue and haematopoietic (blood-forming) elements in various proportions [6].

In the more common human adrenal myelolipoma a hyperintensity in T1- and T2-weighted sequences may lead to the suspicion of a myelolipoma [8]. The haematopoietic tissue is hypointense in T1-weighted images and hyperintense in T2-weighted images. The nonuniform admixture of fat and marrow elements also may result in an inhomogenous appearance on T2-weighted images [8-11].

Idiopathic sterile pyogranulomatous inflammation, leading to a T2-weighted hyperintensity of the vertebral body and spinal cord is an important differential diagnosis to the MRI findings and is known to cause spinal cord compression in Miniature Dachshunds [12,13]. Another differential to the MRI findings is an extramedullary haematopoietic tumor which may look identical to an extraadrenal myelolipoma, and has been described to cause spinal cord compression in humans [14].

To the authors’ knowledge there are only two published cases of epidural myelolipoma in dogs [6,7]. Only one published case of an epidural myelolipoma with MRI in a dog exists [6], but without a fat suppression sequence.

Interestingly, there are some similarities between the cases described and the case presented herein: the dogs were all sled dogs of the same age and gender, a male Siberian husky (11 years), a male Alaskan Malamute (13 years), and the presented male Husky-cross (11.5 years) and therefore with a close genetic ancestry. Together with the Samoyed these breeds descend from the original sled dog. The lesion sites were also similar, namely at the level of L1 and L2, and from Th13 to L3 in the Alaskan Malamute and Husky-cross, and in the Siberian husky, respectively.

## Conclusion

Myelolipoma can occur epidurally and cause signs of proprioceptive ataxia and spinal hyperaesthesia. MRI is particularly useful to determine the underlying cause of this myelopathy. Particularly the fat suppression sequence is beneficial to identify the mass as adipose tissue, which predominates this lesion. Myelolipoma should be a differential diagnosis in cases of extradural spinal cord compression.

Everything performed was wanted and approved by the owner of the dog and no experimental research has been performed.

## Competing interest

The authors declare that they have no competing interests.

## Authors’ contributions

MVH performed the clinical workup of the patient and drafted the manuscript. DCL performed the diagnostic imaging. CL and VH carried out the post-mortem examination. VMS was involved in the clinical workup and drafting the manuscript. All authors read and approved the final manuscript.

## References

[B1] Latimer KS Rakich PM Subcutaneous and hepatic myelolipomas in four exotic birds Vet Pathol 1995 32 84 87 10.1177/030098589503200117 7725606

[B2] Valli VE Jacobs RM Parodi AL Vernan W Histological classification of the hematopoietic tumors of domestic animals WHO international classification of tumors of domestic animals, Volume 8 2002 2 Washington, DC: Armed Forces Institute of Pathology 27

[B3] Meuten DJ Cullen JM Popp JA Meuten DJ Tumors of the Liver and Gall Bladder. & Capen CC: Tumors of the Endocrine Glands Tumors in Domestic Animals 2002 4 Iowa: Iowa State Press 483 508 607–696

[B4] Morandi F Mays JL Newman SJ Adams WH Imaging diagnosis - bilateral adrenal adenomas and myelolipomas in a dog Vet Radiol Ultrasound 2007 48 246 249 10.1111/j.1740-8261.2007.00237.x 17508512

[B5] Rao P Kenney PJ Wagner BJ Davidson AJ Imaging and pathological features of myelolipoma RadioGraphics 1997 17 1373 1385 9397452 10.1148/radiographics.17.6.9397452

[B6] Ueno H Miyake T Kobayashi Y Yamada K Uzuka Y Epidural spinal myelolipoma in a dog J Am Anim Hosp Assoc 2007 43 132 135 17339292 10.5326/0430132

[B7] Newman SJ Inzana K Chickering W Extradural Myelolipoma in a dog J Vet Diagn Invest 2000 12 71 74 10.1177/104063870001200115 10690782

[B8] Ferrozzi F Bova D CT and MR demonstration of fat within an adrenal cortical carcinoma Abdom Imaging 1995 20 272 274 10.1007/BF00200415 7620426

[B9] Kammen BF Elder DE Fraker DL Siegelman ES Extraadrenal myelolipoma: MR imaging findings Am J Roentgenol 1998 171 721 723 10.2214/ajr.171.3.9725304 9725304

[B10] Kelekis NL Alexopoulou E Brountzos EN Ladis V Boussiotou A Kelekis DA Giant adrenal myelolipoma with minimal fat content in a patient with homozygous beta-thalassemia: appearance on MRI J Magn Reson Imaging 2003 18 608 611 10.1002/jmri.10401 14579404

[B11] Musante F Derchi LE Bazzocchi M Avataneo T Gandini G Pozzi Mucelli RS MR imaging of adrenal myelolipomas J Comput Assist Tomogr 1991 15 111 114 10.1097/00004728-199101000-00017 1987178

[B12] Aikawa T Yoshigae Y Kanazono S Epidural idiopathic sterile pyogranulomatous inflammation causing spinal cord compression injury in five miniature dachshunds Vet Surg 2008 37 594 601 10.1111/j.1532-950X.2008.00436.x 19134111

[B13] Nishida H Tanaka H Kitamura M Hatoya S Sugiura K Inaba T Nakayama M Three cases of idiopathic sterile pyogranulomatous inflammation of epidural fat in miniature dachshunds J Vet Med Sci 2012 74 1071 1074 10.1292/jvms.12-0028 22467074

[B14] Lau SK Chan CK Chow YY Cord compression due to extramedullary hematopoiesis in a patient with thalassemia Spine 1994 19 2467 2470 10.1097/00007632-199411000-00019 7846603

